# Successful Management of Blue Rubber Bleb Nevus Syndrome (BRBNS) with Sirolimus

**DOI:** 10.1155/2018/7654278

**Published:** 2018-10-08

**Authors:** Ugochi O. Ogu, Ghada Abusin, Rolla F. Abu-Arja, Janice M. Staber

**Affiliations:** ^1^Division of Hematology, Department of Oncology, Montefiore Medical Center, Bronx, NY, USA; ^2^Division of Pediatric Hematology/Oncology, Department of Pediatrics, University of Michigan, Ann Arbor, MI, USA; ^3^Division of Pediatric Hematology/Oncology/BMT, Nationwide Children's Hospital, Columbus, OH, USA; ^4^Division of Pediatric Hematology/Oncology, Stead Family Department of Pediatrics, University of Iowa Carver College of Medicine, Iowa City, IA, USA

## Abstract

Blue rubber bleb nevus syndrome (BRBNS) is a rare disease with vascular malformations in several systems of the body, most commonly the skin and gastrointestinal tract. Bleeding from the gastrointestinal (GI) tract is a major complication, which may lead to chronic iron deficiency anemia and the need for frequent blood transfusions due to ongoing gastrointestinal blood loss. In this case report, we describe a now 19-year-old female with BRBNS who required six blood transfusions per year and after starting sirolimus is symptom- and transfusion-free.

## 1. Introduction

Vascular anomalies can be divided into two broad categories, according to the International Society for the Study of Vascular Anomalies (ISSVA) system: vascular malformations and vascular neoplasms [1]. Vascular malformations include slow-flow malformations (with venous, capillary and/or lymphatic components) and fast-flow malformations (with arterial components); while vascular neoplasms undergo mitosis and include such lesions as infantile hemangioma, congenital hemangioma, kaposiform hemangioendothelioma, tufted angioma, hemangiopericytoma, and angiosarcoma [2]. Blue rubber bleb nevus syndrome (BRBNS) is primarily considered a slow-flow venous malformation, although there has been a single case report which includes a lymphatic component [3]. BRBNS usually presents in infancy and childhood and rarely in adulthood [3]. Lesions are blue, rubbery, and compressible, and they occur in several organ/systems and most commonly occur within the skin and gastrointestinal (GI) tract [4].

## 2. Case Description

**Figure 1:**
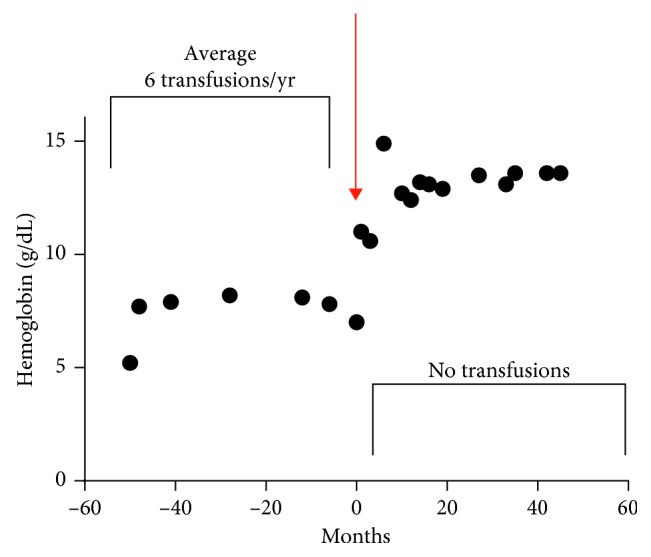
Hemoglobin concentration before and after Sirolimus therapy.

A Hispanic female initially presented at three years of age with a history of oropharyngeal bleeding since birth and diffuse skin vascular malformations. Upper and lower GI endoscopies revealed multiple vascular anomalies throughout the entire tract. She was subsequently diagnosed with blue rubber bleb nevus syndrome based on clinical and endoscopic findings. Due to GI bleeding, chronic iron deficiency anemia, and the increased need for blood transfusions, she underwent surgical removal of multiple blebs from her stomach, small intestine, and colon. In addition, she underwent a right colectomy, gastrostomy for tube feedings, and a tracheostomy due to multiple tracheal lesions. At the age of 6.5 years, she was referred to pediatric hematology for the management of anemia. She had long-standing iron deficiency anemia due to significant blood loss from GI bleeding, despite previous RBC transfusions, and intravenous iron therapy. To help control bleeding, she underwent frequent sclerosing therapies to multiple lesions, including the pericervical lesions. An oral aminocaproic acid (Amicar) trial of 10 days duration was not successful to reduce GI bleeding. Despite the above interventions, she remained severely anemic (hemoglobin levels 5.2 gm/dL to 7 gm/dL) and required frequent blood transfusions, as often as every 1–4 months. Due to the significant GI bleeding, her stools were black, tarry, often with bright red blood, occurring 3–4 times a week. At age 15, a trial of daily sirolimus therapy was initiated, based on a case report by Yuksekkaya et al. [4], at a dose of 0.05 mg/kg/dose and levels followed with a target range of 5–10 ng/ml. Within 2 months of initiating sirolimus therapy, she experienced cessation of hematochezia and melena, and her hemoglobin has since remained above 11 g/dl (Figure 1). She is now over 60 months into therapy, remains without anemia, and has not required further blood transfusions. She remains mildly iron deficient to date, most likely due to decreased ability to adequately absorb oral iron due to the blebs and prior GI surgery. No adverse drug reactions have occurred.

## 3. Discussion

Blue rubber bleb nevus syndrome is a rare congenital disorder with hallmarks of venous malformations on the skin and viscera. The skin and soft tissue lesions rarely cause debilitating disease and are mostly a cosmetic concern [4]. In contrast, the GI lesions are a major cause of morbidity. Patients usually develop severe chronic iron deficiency anemia, requiring multiple transfusions due to persistent GI losses [4].

To date, there is no curative treatment for BRBNS. Management options that have been attempted include iron therapy, blood transfusions, surgical interventions, and pharmacologic agents [4]. Iron therapy and blood transfusions have been employed to alleviate anemia from GI losses. Surgery has been used to eradicate blebs from the skin, soft tissue, or GI tract; however, the blebs eventually recur. Other modalities including laser photocoagulation and sclerotherapy have been applied with limited success. Pharmaceutical agents such as propranolol, octreotide, corticosteroids, interferon alpha, thalidomide, antifibrinolytics, and most recently sirolimus have also been utilized. These have been used based on extrapolation of their efficacy in infantile hemangiomas and other vascular anomalies.

Sirolimus is an immunosuppressant drug that has both antiangiogenic and antineoplastic properties. Its mechanism of action is via pathway inhibition of the mammalian target of rapamycin (mTOR), a serine/threonine kinase regulated by phosphoinositide-3-kinase (PI3K) [5]. It has been used successfully in the management of several vascular anomalies such as kaposiform hemangioendothelioma, tufted angioma, and lymphatic malformations [1]. The first case report describing response of BRBNS to sirolimus was published in 2012 by Yussekkaya et al. [4]. Following that there have been several other reports also describing response to sirolimus [3, 6–14].  Table 1 details the various reports in the literature so far that have described the use of sirolimus for BRBNS.

The exact mechanism of action of sirolimus in BRBNS remains unclear, but proposed mechanisms of action include inhibition of ligand-binding-induced signaling through VEGFR-3 (vascular endothelial growth factor receptor-3) on lymphatic endothelial surface, which would normally result in activation of the PI3K/Akt/mTOR pathway [3, 5]. In addition, c-kit (stem cell growth factor receptor) expression from small venous vessels has been described [15] and has been proposed as a possible mechanism of action, given that c-kit is a tyrosine kinase upstream of mTOR [3].

There exists a dilemma as to what constitutes appropriate duration of therapy. Our patient continues on sirolimus at a dose of 1.2 mg/day (0.024 mg/kg/dose), with target trough level of 2–4 ng/mL and has not experienced any side effects. Her hemoglobin and symptoms remained controlled. Of note, when sirolimus was held for a surgical procedure due to concerns for postsurgical wound healing, her GI bleeding returned within 3 days of discontinuation. Further studies are needed to determine if sirolimus can be safely discontinued, without disease relapse.

In conclusion, sirolimus may be used in management of patients with BRBNS. Our case report describes resolution of GI bleeding and obviation of the need for multiple blood transfusions following initiation of sirolimus therapy. We propose this as an alternative therapy for the treatment of symptomatic BRBNS, especially when other conventional therapies have proved to be unsuccessful.

## Figures and Tables

**Table 1:** Articles on BRBNS with Sirolimus therapy.

Article authors	Article title	Article journal	Article year	Patient	Response to Sirolimus	Sirolimus dose	Sirolimus level	Time to improvement
Yuksekkaya H, Ozbek O, keser M, Toy H [4]	Blue rubber bleb nevus syndrome: successful treatment with Sirolimus	*Pediatrics*	2012	8 yo F; GI bleeding, not responsive to prednisolone, propranolol, aminocaproic acid, and alpha-interferon therapy	Hemoglobin improved from 7 gm/dL to 14 gm/dL (with iron supplement);	0.05–0.1 mg/kg	1–5 ng/mL	2 months. Symptoms returned if sirolimus discontinued
Taddio A, Benelli E, Pierobon C, Martelossi S, Berti I, Ventura A [6]	From skin to gut	*J Pediatr*	2013	3 yo M; severe iron deficiency anemia, large subcutaneous swelling on right ankle, and multiple skin lesions	Hemoglobin improved from 6.4 gm/dL to 'stable'	not available (NA)	NA	6 months
Ozgonenel B, Martin A [9]	Low-dose sirolimus controls recurrent iron deficiency in a patient with blue rubber bleb nevus syndrome	*Pediatr Blood Cancer*	2015	18 yo F; GI bleeding and iron deficiency anemia	Hemoglobin improved from 5 gm/dL to ∼14 gm/dL	1.6 mg/m^2^/day divided BID; reduced to 0.6 mg/m^2^/day	10–15 ng/mL, then goal levels reduced <2.0–3.1 ng/mL	“after starting sirolimus”
Ferrés-Ramis L, knöpfel N, Salinas-Sanz J, Martín-Santiago A [8]	Rapamycin in the treatment of blue rubber bleb nevus syndrome	*Actas Dermo-Sifiliográficas (English Edition)*	2015	8 yo with congenital cutaneous and GI vascular malformations	Decreased size of lesions, normalized hemoglobin	Initial dose 0.05 mg/kg, reduced to 0.02 mg/kg		Within a month
Warner B, Butt A, cairns S [7]	Sirolimus is a successful treatment for recurrent iron deficiency anemia in blue rubber bleb nevus syndrome	*J Pediatr Gastroenterol Nutr*	2015	18 yo M; hemangioma, tracheostomy, anemic; thalidomide—discontinued due to side effects; multiple bowel resections to remove vascular malformations	Hemoglobin 6.9 gm/dL, anemia resolved	4 mg daily		
Salloum R, Fox CE, Alvarez-Allende CR, et al. [3]	Response of blue rubber bleb nevus syndrome to sirolimus treatment.	*Pediatr Blood Cancer*	2016	2–16 yo, cutaneous, GI tract, visceral and muscular lesions	Decreased size of lesions, decreased pain, normalized hemoglobin	0.8 mg/m^2^ every 12 hour	dose titrated to target trough level between 10 and 13 ng/mL	less than 3 months
Cardoso H, Dias JA, Silva M, et al. [10]	Gastrointestinal: successful treatment with sirolimus of a patient with blue rubber bleb nevus syndrome	*J Gastroenterol Hepatol*	2016	19 yo M; multiple visceral, muscular, and subcutaneous vascular lesions, complicated with chronic local pain and GI bleeding requiring RBC transfusion (total of 74); treatment with thermal argon ablation and sclerosis and segmental jejunoileal surgical resections, propranolol and ferric carboxymaltose	improved blood loss, asthenia, and decreased size of lesions; hemoglobin improved by 6 gm/dL	2 gm/day		about 5 months
Ünlüsoy Aksu A, Sari S, Eğritaş Gürkan Ö, Dalgiç B [11]	Favorable response to sirolimus in a child with blue rubber bleb nevus syndrome in the gastrointestinal tract	*J Pediatr Hematol Oncol*	2017	11 yo M with vascular malformation in GI tract	Normalized hemoglobin in 2.5 months, decreased lesions in 5 months	0.1 mg/kg/d	1–5 ng/mL	2.5–5 months
Akyuz C, Susam-Sen H, Aydin B [12]	Blue rubber bleb nevus syndrome: promising response to sirolimus	*INDIAN Pediatr*	2017	6 yo F with skin lesions, GI tract and consumptive coagulopathy (platelets 77K, fibrinogen 104 mg/dL, d-dimer >40 mg/dL); oral steroids without success; bleeding requiring transfusions (hemoglobin 6.1 gm/dL)	improved size and number of lesions, no further GI bleeding or anemia	1.6 to 2 mg/m^2^/day	5–12 ng/mL	Less than 1 month. Disconintued sirolimus and at 4 months off therapy- no evidence of microscopic blood in stool and normal hemaglobin levels, stable lesions
Wang KL, Ma SF, Pang LY, Zhang MN, Hu LY, Liu MJ, Zou LP [13]	Sirolimus alternative to blood transfusion as a life saver in blue rubber bleb nevus syndrome	*Medicine (Baltimore)*	2018	12 yo F with multiple hemangiomas on head and neck, limbs and trunk, tip of tongue and digestive tract. Severe anemia, requiring red cell transfusion every 2 weeks. Mutation in exon 15 of TEK gene	Improved hemoglobin, skin and digestive tract hemangiomas, no further transfusions	1 mg/m^2^/d, average 0.7 mg/d	6.2–11.89 ug/L	1 month
Kizilocak H, Dikme G, celkan T [14]	Sirolimus experience in blue rubber bleb nevus syndrome	*J Pediatr Hematol Oncol*	2018	Four children, ages 4–15 years. Three with GI lesions, one with respiratory tract lesions	Normalized hemoglobin, decreased pain, decreased size of lesions	1.2 mg/m^2^/d		2 months

## Abbreviations

BRBNS: blue rubber bleb nevus syndrome
GI: gastrointestinal
mTOR: mammalian target of rapamycin
ISSVA: International Society for the Study of Vascular Anomalies
PI3K: phosphoinositide-3-kinase.
